# Growth Hormone-Releasing Hormone in Diabetes

**DOI:** 10.3389/fendo.2016.00129

**Published:** 2016-10-10

**Authors:** Leonid E. Fridlyand, Natalia A. Tamarina, Andrew V. Schally, Louis H. Philipson

**Affiliations:** ^1^Department of Medicine, Kovler Diabetes Center, The University of Chicago, Chicago, IL, USA; ^2^VA Medical Center, Miami, FL, USA; ^3^Department of Pathology and Medicine, Division of Endocrinology and Hematology-Oncology, Sylvester Comprehensive Cancer Center, University of Miami Miller School of Medicine, Miami, FL, USA; ^4^Department of Pediatrics, The University of Chicago, Chicago, IL, USA

**Keywords:** diabetic complications, GLP-1, islet, insulin, pancreatic beta-cell

## Abstract

Growth hormone-releasing hormone (GHRH) is produced by the hypothalamus and stimulates growth hormone synthesis and release in the anterior pituitary gland. In addition, GHRH is an important regulator of cellular functions in many cells and organs. Expression of GHRH G-Protein Coupled Receptor (GHRHR) has been demonstrated in different peripheral tissues and cell types, including pancreatic islets. Among the peripheral activities, recent studies demonstrate a novel ability of GHRH analogs to increase and preserve insulin secretion by beta-cells in isolated pancreatic islets, which makes them potentially useful for diabetes treatment. This review considers the role of GHRHR in the beta-cell and addresses the unique engineered GHRH agonists and antagonists for treatment of type 2 diabetes mellitus. We discuss the similarity of signaling pathways activated by GHRHR in pituitary somatotrophs and in pancreatic beta-cells and possible ways as to how the GHRHR pathway can interact with glucose and other secretagogues to stimulate insulin secretion. We also consider the hypothesis that novel GHRHR agonists can improve glucose metabolism in Type 2 diabetes by preserving the function and survival of pancreatic beta-cells. Wound healing and cardioprotective action with new GHRH agonists suggest that they may prove useful in ameliorating certain diabetic complications. These findings highlight the future potential therapeutic effectiveness of modulators of GHRHR activity for the development of new therapeutic approaches in diabetes and its complications.

## Introduction

Type 2 diabetes mellitus (T2DM) is an important metabolic disease affecting almost 30 million Americans with an estimated $250 billion lost yearly, due to effects of morbidity and mortality on total medical costs and lost wages. T2DM is associated with a progressive decline in insulin secretion by pancreatic beta-cells in the face of insulin resistance (1). Despite its importance, we do not fully understand the complex interplay of molecular signals and signal transduction events that control beta-cell functionality and survival. This limits our ability to develop novel approaches for prevention and treatment of diabetes.

The beta-cell membrane contains a profusion of G-protein coupled receptors (GPCRs) that are critical for the regulation of insulin secretion by hormones and neurotransmitters (2–4). Growth hormone-releasing hormone (GHRH) is an important regulator not only of growth hormone secretion but also of a variety of cellular functions in many cells and organs. Expression of GHRH G-protein coupled receptor (GHRHR) has been demonstrated in different peripheral tissues and cell types, including pancreatic islets (5, 6).

Recent studies demonstrate a novel ability of GHRH analogs to increase and preserve insulin secretion by beta-cells in islets and diabetic mice (7, 8) that makes them potentially useful for treatment of T2DM. Remarkable results from the study of new GHRH agonists in wound healing and cardiovascular performance could also provide novel treatments in patients with diabetes (5, 7, 9). This review addresses the possible role of GHRHR and its unique engineered agonists and antagonists for treating diabetes and its complications.

## GHRH and Its Analogs

Hypothalamic growth hormone-releasing hormone is one of the “humoral factors” that is critical for growth hormone secretion. The discovery of hypothalamic hormones, such as thyrotropin-releasing hormone, luteinizing hormone-releasing hormone (also known as gonadotropin-releasing hormone), which regulate the secretion of anterior pituitary hormones led to the awarding of the Nobel Prize (1977) to one of us (Andrew V. Schally) (10). GHRH, expressed in the arcuate nucleus of the hypothalamus and released into portal vasculature, directly stimulates growth hormone synthesis and secretion from the pituitary somatotropes by activating the corresponding GHRH receptors (5, 11). GHRHR is present in several other tissues, such as myocardium, lymphocytes, testes, ovaries, skin, and pancreas and is involved in a variety of biological processes (5). The roles of GHRHR in other cells and tissues continue to be explored. In addition, GHRHR have been detected in various tumor cells and in some stem cells (5, 6).

It should be noted that GHRH undergoes rapid enzymatic degradation in blood. Dipeptidylpeptidase IV inactivates the active form of GHRH in blood to its more stable inactive metabolite GHRH(3-44)-NH2 (12, 13). For this reason, concentration of active GHRH (that is produced in the hypothalamus) in blood may be insignificant, and so without significant influence on organs beyond the pituitary somatotropes. Interestingly, inhibitors to dipeptidylpeptidase IV are in widespread use now for type 2 diabetes treatment to increase GLP-1 concentration in blood (14). These agents should also lead to increased GHRH blood concentration. However, this interesting possibility and the effect of GHRH on various target tissues where the GHRHR is expressed have not been investigated.

Accumulating evidence also suggests that, in addition to the neuroendocrine action of GHRH, extrahypothalamic GHRH has been implicated in many peripheral actions *via* autocrine/paracrine mechanisms. Exogenous GHRH can regulate proliferation, survival, apoptosis, and differentiation in several tissues and cell types (5, 15).

The GHRHR is a member of the class II B GPCR family, which couples predominantly to the Gs-adenylate cyclase-cAMP signaling pathway. Peptide hormones that activate class II GPCRs include GHRH, secretin, glucagon-like peptides, gastric-inhibitory peptide (GIP), pituitary adenylate cyclase-activating peptide, corticotropin-releasing hormone, vasoactive intestinal peptide, parathyroid hormone, and calcitonin-related peptides (16, 17).

**Figure 1:**
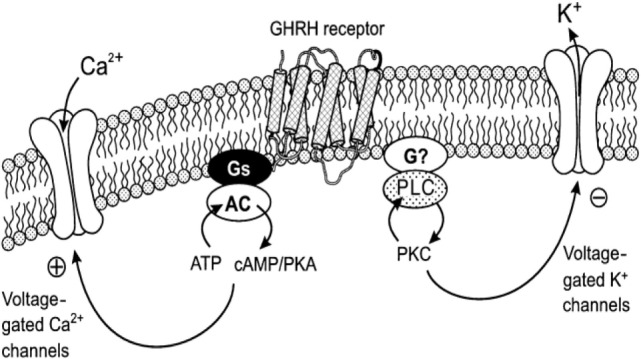
**Mechanism of the action of GHRH on Ca^2+^ and K^+^ channels: coupling with protein kinase A (PKA) and protein kinase C (PKC) systems**. This diagram illustrates the coupling of the Ca^2+^ and K^+^ channels with GHRH receptors. cAMP–PKA system mediates the action of GHRH on voltage-gated Ca^2+^ currents, and the PKC system is essential for the action of GHRH on voltage-gated K^+^ currents in somatotropes. AC, adenylyl cyclase; PLC, phospholipase C. Reprinted by permission from Macmillian Publishers Ltd., from Ref (23), Figure 11.

The mechanism of the acute action of GHRH on the pituitary somatotrope to increase growth hormone synthesis and secretion has been studied (Figure 1). Binding of GHRH to its receptor activates a stimulatory G protein, which activates adenylyl cyclase to produce cAMP, leading to activation of protein kinase A (PKA). This stimulates an influx of calcium, most likely through plasma membrane depolarization, and activation of voltage-sensitive Ca^2+^ channels. Increased Ca^2+^ and cAMP stimulates the growth hormone exocytosis process (18–21). For example, forskolin (adenylate cyclase activator) increases Ca^2+^ influx in somatotrophs, and inhibition of phosphodiesterase increases the electrical activity of somatotrophs confirming the relevance of cAMP in GHRH action (22). Regulated secretion of growth hormone involves movement of secretory vesicles along microtubules, transient “docking” in the cell membrane, and subsequent release of vesicles (21).

In pituitary somatotrophs, upon binding of the ligand GHRH to the GHRHR, the activated second messengers include not only the adenylate cyclase–cAMP–PKA and Ca^2+^-calmodulin but also inositol phosphate–diacylglycerol–protein kinase C (PKC), L-type calcium channels, and arachidonic acid–eicosanoic pathways as well, these ultimately result in the stimulation of growth hormone production and secretion (23–25). Increased cAMP also stimulates PKA to activate the cAMP response element-binding protein (CREB), which stimulates GHRHR gene transcription.

It is also likely that GHRH function relates to the ability to stimulate somatotroph cell proliferation. The activation of MAP kinase and ERK phosphorylation has been observed in the pituitary in a cAMP/PKA/PKC-dependent manner (26, 27). Alternatively, GHRH can stimulate the Ras/MAPK *via* βγ-subunits, to promote cell growth (26). In the myocardium, GHRHR-mediated inhibition of apoptosis involves modulation of ERK1 and ERK2 and PI3K−Akt signaling because ERK1/2- and PI3K/Akt-specific inhibitors abolished these effects (28).

Numerous high affinity and high specificity agonists and antagonists of GHRHR have been developed (9, 29–31). There are remarkable results from studies of GHRH agonists for wound healing, cardioprotective action, and protection from pneumolysin-induced pulmonary permeability edema (5, 7, 9, 32, 33). On other hand, GHRHR antagonists show prominent effects in augmenting apoptosis and decreasing proliferation of multiple types of cancer cells (30, 34, 35).

## Effects of GHRH and Relevant GHRHR Agonists in Pancreatic Beta-Cell and Islets

Insulin is produced by pancreatic beta-cells in the islets of Langerhans. GHRH receptors have been described in primary as well as clonal pancreatic beta-cells (insulinoma cells) and isolated islets (7, 8, 36, 37). Human GHRH can acutely stimulate insulin secretion from isolated rodent islets and dispersed beta-cells (38, 39) and from perfused dog pancreas (40). Intravenous injection of human GHRH to rats increased plasma concentration of insulin being released into the hepatic portal vein (39). In another functional assay, pretreatment with synthetic GHRH analogs improved the engraftment and the metabolic function of islets, following transplantation to streptozotocin (STZ)-induced diabetic mice (36). Pretreatment of rat islets with the GHRH agonist, JI-36, significantly enhanced graft function by improving glucose tolerance and increasing beta-cell insulin reserve in rats (41). Novel high affinity and high specificity agonists of GHRHR improve insulin secretion and preserve beta-cells and islets in lethality assays (7, 8). Based on these findings, GHRH and its corresponding receptor hold promising therapeutic potential for improving beta-cell function and possibly treating T2DM.

Interestingly, the discovery of GHRH was due in part to the recognition of ectopic GHRH secretion from human pancreatic islet tumors causing ectopic acromegaly (42–45). GHRH was, thus, found in human pancreatic tumor tissue extracts, leading to its structural elucidation (44). These data suggest that perhaps GHRH is expressed at low levels by pancreatic islet cells and possibly during development also at low levels. This suggests that GHRH may be part of a paracrine system in islets, but this possibility has not yet been investigated. It is also possible that the GHRHR exerts an influence on cell function even without receptor activation through some tonic receptor function.

Despite these advances, the details of GHRHR expression, signaling pathways, and function in pancreatic islet cells have not been fully elucidated. We will consider the possible mechanisms of regulation of insulin secretion as well as mechanisms relating to beta-cell proliferation to evaluate the possible roles of GHRHR activation.

**Figure 2:**
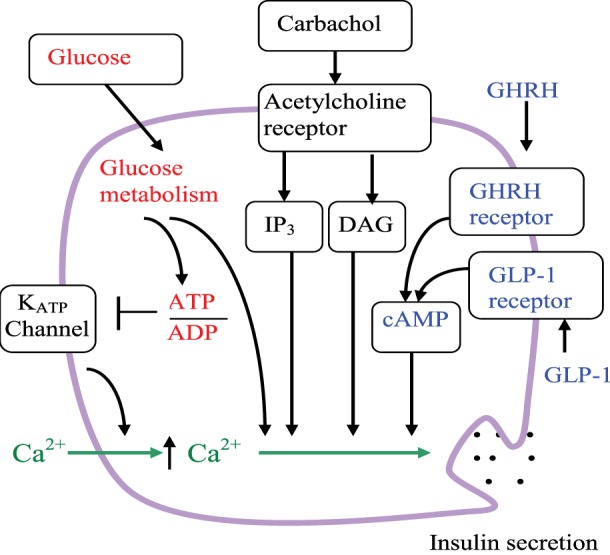
**Schematic of the beta-cell signaling pathways and hypothetical role of GHRHR**. Arrows indicate activation or an increase in concentration. The line ending with a bar indicates inhibition or closure. Glucose metabolism increases the ATP/ADP ratio, leading to the closure of K_ATP_ channels, reduction of K^+^ efflux, membrane depolarization, increase of intracellular Ca^2+^, and insulin secretion. Glucose also leads to insulin secretion through amplifying pathways that are independent of K_ATP_ channels. Carbachol stimulation enhanced insulin secretion *via* the acetylcholine (muscarinic) receptor and phospholipase C pathways. IP_3_ is inositol 1,4,5-trisphosphate, DAG is diacylglycerol. As depicted, GLP-1 and GHRH both enhance insulin secretion *via* the cAMP pathway.

The primary role of pancreatic beta-cells is to regulate metabolism by sensing changes in blood glucose concentration and responding by secreting precisely regulated amounts of insulin. The action of hormones and neurotransmitters contribute to such signaling and amplify the glucose-stimulated insulin secretion (GSIS) (46) (Figure 2). GSIS is Ca^2+^-dependent and is regulated by metabolic signals generated by glucose catabolism. Glucose-dependent signal transduction begins with uptake of glucose into beta-cells *via* the GLUT2 transporter. Cytoplasmic glucose molecules are rapidly phosphorylated by glucokinase and converted to pyruvate in the cytosol *via* the glycolytic pathway, then oxidized within the mitochondria *via* the tricarboxylic acid cycle and oxidative phosphorylation pathways, respectively. Glucose catabolism generates ATP. The membrane potential of beta-cells is controlled by K_ATP_ channels. Under basal conditions, sufficient K_ATP_ channels are open so that the plasma membrane is hyperpolarized. Blocking K_ATP_ channels by an ATP-dependent mechanism initiates plasma membrane depolarization that opens voltage-gated Ca^2+^ channels; Ca^2+^ enters the beta-cell from the extracellular milieu through of these channels and increases cytoplasmic Ca^2+^. Glucose-induced increases in cytoplasmic Ca^2+^ and insulin secretion are directly correlated (46, 47).

The beta-cell membrane contains a profusion of GPCRs that are implicated in regulation of insulin secretion by hormones and neurotransmitters. GPCRs may have complimentary or antagonistic actions on insulin secretion (2–4). For example, a stimulation of insulin secretion by food begins with a “cephalic” phase due to sensory stimulation by sight and taste of food. This is largely mediated by the release of acetylcholine from nerves innervating pancreatic islets. The subsequent cholinergic stimulation *via* the muscarinic (acetylcholine) GPCR leads to an activation of the phospholipase C (PLC) pathway (48, 49).

Incretin hormones also play a critical role in insulin secretory response following meal ingestion. These hormones have significant influence on GSIS primarily through activation of the cAMP pathway that also leads to plasma membrane depolarization and an increase in cytoplasmic Ca^2+^ (50, 51). For example, glucagon-like peptide I (GLP-1) is one such potent incretin hormone activating the GPCR alpha(s) that can increase cAMP and activate the PKA pathway in beta-cells. GLP-1 agonists (and DPP4 inhibitors to prolong the half-life of endogenous incretins) have been successfully adopted for T2DM treatment (3, 50).

Growth hormone-releasing hormone and the incretin hormones such as GLP-I and GIP belong to the same class of the structurally related hormones that activate class B GPCRs. As discussed above, these incretin hormones activate the Gs-adenylate cyclase-cAMP signaling pathway, that is, this mechanism can be identical to that of GHRHR in somatotroph cells (see Figures 1 and 2). Interestingly, GHRHR agonists significantly increased the levels of cellular cAMP in rat beta-cell line (INS-1) (7). For this reason, it is reasonable to conclude that GHRHR agonists also employ a cAMP-based signaling mechanism and, therefore, they would have beneficial effects on insulin secretion and beta-cell survival (7). There is also a possibility that activation of cAMP pathway can lead to an increase in cytoplasmic Ca^2+^ concentration that can activate PLC and correspondingly activate this pathway [see, for example, Ref. (4)].

Interestingly, activation of the cAMP pathway in beta-cells by the incretin hormones leads to increased insulin secretion in part due to plasma membrane depolarization and an increase in cytoplasmic Ca^2+^ [for review see Ref. (4, 52)]. Mechanisms by which GHRH increases growth hormone-release also include plasma membrane depolarization and an increase in cytoplasmic Ca^2+^ (see above) that also point to significant similarities between the mechanisms of GLP-1 in beta-cells and the mechanisms of GHRH in the pituitary somatotropes.

Activation of GHRH receptors may also lead to activation of gene transcription, proliferation, and survival in beta-cells. For example, the mechanisms of beta-cell proliferation and survival include ERK and Akt signaling pathways (53). GHRHR agonists activate these pathways in various cell types (see above). Experiments in rat insulinoma cells (INS-1) showed that the GHRH agonist MR-409 significantly increased cell proliferation and induced activation of ERK and Akt pathways (7).

GHRH G-protein coupled receptor agonists significantly increased the levels of the phosphorylation of CREB in rat beta-cell line (INS-1) (7) as well as in somatotrophs, where GHRHR agonists can stimulate growth hormone gene transcription (see above). This may also be similar to one of the mechanisms of GLP-1. GLP-1 has also been shown to promote beta-cell proliferation and survival in rodents by activating ERK and Akt pathways (50). cAMP induced by GLP-1 caused elevated phosphorylation of CREB/activating-transcription-factor-1 in insulin-secreting beta-cells (54). However, the exact mode(s) of GHRHR signaling in the pancreatic islets and the most important mechanisms of stimulation of insulin secretion and/or beta-cell survival are unknown.

Causal interrelationships between GHRHR agonists and the variety of GPCRs in beta-cells and the role of such networks in insulin secretion are unknown. We have recently employed our general beta-cell mathematical modeling approach for beta-cell GPCRs for a comparison of action of GPCRs for GLP-1 and GIP (4). Both of them couple predominantly to the Gs-adenylate cyclase-cAMP signaling pathway. Based on those models, we suggest that GHRHR agonists can have a similar role as GIP in its interaction with GLP-1. In this case, GHRHR agonists can act in a competitive manner with GLP-1 in their mechanisms of stimulating insulin secretion. This testable hypothesis remains to be directly demonstrated.

There are several possible ways as to how pancreatic beta-cell GHRH signaling can have implications in T2DM treatment. One of the root causes of T2DM is the altered signaling system in beta-cells, which leads to decreased insulin production and exocytosis. Our previous published data and that of several other groups suggest that some signaling systems in insulin-secreting cells are damaged or attenuated in diabetic states. “Diabetic conditions” – such as hyperglycemia and hyperlipidemia – can lead to the loss of the GLP-1 receptor (GLP-1R) from the cell surface and, thereby, impair GLP-1 signaling, which may underlie the reduced clinical efficacy of GLP-1R activators (50, 51). GHRHR agonists can have beneficial effects under these conditions since these agonists activate the same cAMP pathway as GLP-1R, assuming the GHRHR is not also downregulated. Therefore, we can hypothesize that glycemic insensitivity to GLP-1R agonists in T2DM can be improved by simultaneous or sequential application of GHRHR agonists, thus replacing a possible deficit of GLP-1R.

*Ex vivo* treatment of isolated islets with GHRH agonists may also improve results of islet transplantation in animal models. Preconditioning of encapsulated pancreatic islets with GHRHR agonists significantly enhanced graft function by improving glucose tolerance and increasing beta-cell insulin reserve in diabetic rats (41). This effect is of sufficient interest to further examine it in human islet transplantation.

Interestingly, GHRH stimulated and GHRH antagonist inhibited the expression of the major antioxidant enzymes in the LNCaP human prostate cancer line (55). Additional expression of the major antioxidant enzymes may have additional benefits in T2DM (56) as well as T1D (57), and this may be another potentially beneficial effect of GHRHR agonists in both major types of diabetes.

Based on these studies, we suggest that GHRHR analogs have the potential to enhance beta-cell function, proliferation, and survival *in vivo*. Further studies with human islets and beta-cells will help determine if the GHRHR expression levels and signaling systems are similar in human and rodent models.

## Diabetes and Activity of GHRH Beyond Beta-Cells

Remarkable results from the study of new GHRH agonists in wound healing and cardiovascular performance could also suggest novel treatments in patients with diabetes or perhaps help understand the pathways involved (5, 7, 9). GHRHR antagonists may target certain complications of diabetes, especially in Type 1 diabetes and insulin-dependent T2DM, where insulin production by the beta-cell is at least clinically insignificant. For example, GHRH antagonism may improve some of the lipid, renal, and vascular complications of low insulin-associated diabetes (58). Another potential target for GHRH antagonists could be diabetic retinopathy, which is the main cause of blindness in patients with diabetes and diabetic nephropathy (glomerulosclerosis) (30). Despite remarkable advances in treatment and prevention of these complications, they are still dramatic components of the long-term costs of diabetes.

Gastrointestinal effects are also complications of diabetes. There was upregulation of GHRHR expression in intestinal cells in a mouse model of type 2 diabetes (58). Treatment with the GHRHR antagonist, MIA-602, interfered with GLP-1-dependent diabetes-related dyslipidemia in mice. It also decreased the plasma levels of GLP-1, glucagon, and TRL in these mice (58), which might lead to worsening of diabetes rather than improvement. Cross-talk between the GHRHR antagonist and acetylcholine signaling (M3 receptor) was observed in the aorta, where MIA-602 prevented the diabetes-related block of carbachol-mediated vasodilation (58).

Interestingly, human GHRH can reduce glucagon release from isolated mouse islets (39). We can explain this by the increased insulin secretion that suppresses glucagon secretion in this case [see, for example, Ref. (59)]. However, enhanced glucagon secretion by islet cells in diabetes was also lowered by application of antagonist MIA-602 (58). The decreased glucagon secretion in this case can be explained by cAMP decrease in alpha cells [see, for example, Ref. (60)] through blocking the corresponding GHRH receptor when insulin secretion is insignificant in islets from diabetic animals or humans. Decreased glucagon release by GHRHR antagonists could have a beneficial effect in diabetes through decreasing hepatic glucose production and perhaps decreasing ketogenesis (59, 61).

## Conclusion

This review of recent data with GHRHR agonists shows them to be capable of acutely increasing insulin secretion and enhancing rodent beta-cell proliferation and survival, when administered systemically. On the other hand, the modulators of GHRHR activity may be useful in ameliorating certain complications of diabetes. Studies are currently ongoing to determine the dose and treatment regimes of GHRHR modulators for the treatment of other diseases. The results demonstrate a clear connection of GHRH and its receptor with glucose metabolism and pancreatic beta-cell function. We believe that there is a sound basis for further studies evaluating GHRHR agonists and/or antagonists as promising therapeutic agents for diabetes and its complications.

## Author Contributions

LF, NT, AS, and LP reviewed the literature, designed the work, and, together, wrote and edited the paper.

## Conflict of Interest Statement

The authors have no commercial or financial relationship to disclose that could have influenced the redaction of the present review.

## Abbreviations

GHRH, growth hormone-releasing hormone; GHRHR, GHRH G-protein coupled receptor; GLP-1, glucagon-like peptide 1; GLP-1R, receptor for GLP-1; GSIS, glucose-stimulated insulin secretion; GPCR, G-protein coupled receptor; K_ATP_ channels, ATP-sensitive K^+^ channels; PKA, protein kinase A; PLC, phospholipase C, T2DM, type 2 diabetes mellitus.

## References

[1] Kahn SE Cooper ME Del Prato S. Pathophysiology and treatment of type 2 diabetes: perspectives on the past, present, and future. Lancet (2014) 383(9922):1068–83. 10.1016/S0140-6736(13)62154-6 24315620 PMC4226760

[2] Winzell MS Ahren B. G-protein-coupled receptors and islet function-implications for treatment of type 2 diabetes. Pharmacol Ther (2007) 116(3):437–48. 10.1016/j.pharmthera.2007.08.002 17900700

[3] Amisten S Salehi A Rorsman P Jones PM Persaud SJ. An atlas and functional analysis of G-protein coupled receptors in human islets of Langerhans. Pharmacol Ther (2013) 139(3):359–91. 10.1016/j.pharmthera.2013.05.004 23694765

[4] Fridlyand LE Philipson LH. Pancreatic beta cell G-protein coupled receptors and second messenger interactions: a systems biology computational analysis. PLoS One (2016) 11(5):e0152869. 10.1371/journal.pone.0152869 27138453 PMC4854486

[5] Kiaris H Chatzistamou I Papavassiliou AG Schally AV. Growth hormone-releasing hormone: not only a neurohormone. Trends Endocrinol Metab (2011) 22(8):311–7. 10.1016/j.tem.2011.03.006 21530304

[6] Ziegler CG Brown JW Schally AV Erler A Gebauer L Treszl A Expression of neuropeptide hormone receptors in human adrenal tumors and cell lines: antiproliferative effects of peptide analogues. Proc Natl Acad Sci U S A (2009) 106(37):15879–84. 10.1073/pnas.0907843106 19717419 PMC2733863

[7] Zhang X Cui T He J Wang H Cai R Popovics P Beneficial effects of growth hormone-releasing hormone agonists on rat INS-1 cells and on streptozotocin-induced NOD/SCID mice. Proc Natl Acad Sci U S A (2015) 112(44):13651–6. 10.1073/pnas.1518540112 26474831 PMC4640729

[8] Schubert U Schmid J Lehmann S Zhang XY Morawietz H Block NL Transplantation of pancreatic islets to adrenal gland is promoted by agonists of growth-hormone-releasing hormone. Proc Natl Acad Sci U S A (2013) 110(6):2288–93. 10.1073/pnas.1221505110 23345449 PMC3568317

[9] Cai R Schally AV Cui T Szalontay L Halmos G Sha W Synthesis of new potent agonistic analogs of growth hormone-releasing hormone (GHRH) and evaluation of their endocrine and cardiac activities. Peptides (2014) 52:104–12. 10.1016/j.peptides.2013.12.010 24373935 PMC4745889

[10] Schally AV Aspects of hypothalamus regulation of the pituitary gland with major emphasis on its implications for the control of reproductive processes. Nobel lecture, 8 December 1977. In: Lindsten J, editor. Nobel Lectures, Physiology or Medicine 1071-1980. Singapore: Word Scientific Publishing Co (1992). p. 405–38.

[11] Mayo KE Godfrey PA Suhr ST Kulik DJ Rahal JO. Growth hormone-releasing hormone: synthesis and signaling. Recent Prog Horm Res (1995) 50:35–73. 7740167 10.1016/b978-0-12-571150-0.50007-x

[12] Frohman LA Downs TR Heimer EP Felix AM. Dipeptidylpeptidase IV and trypsin-like enzymatic degradation of human growth hormone-releasing hormone in plasma. J Clin Invest (1989) 83(5):1533–40. 10.1172/JCI114049 2565342 PMC303858

[13] Okimura Y Chihara K Abe H Kaji H Kodama H Kita T Plasma disappearance half-time and metabolic clearance rate of exogenous human growth hormone-releasing hormone-(1-44)-NH2 in normal subjects. Endocrinol Jpn (1986) 33(6):875–81. 10.1507/endocrj1954.33.875 3107967

[14] Nauck M. Incretin therapies: highlighting common features and differences in the modes of action of glucagon-like peptide-1 receptor agonists and dipeptidyl peptidase-4 inhibitors. Diabetes Obes Metab (2016) 18(3):203–16. 10.1111/dom.12591 26489970 PMC4785614

[15] Granata R. Peripheral activities of growth hormone-releasing hormone. J Endocrinol Invest (2016) 39(7):721–7. 10.1007/s40618-016-0440-x 26891937

[16] Frohman LA Kineman RD. Growth hormone-releasing hormone and pituitary development, hyperplasia and tumorigenesis. Trends Endocrinol Metab (2002) 13(7):299–303. 10.1016/S1043-2760(02)00613-6 12163232

[17] Martin B Lopez de Maturana R Brenneman R Walent T Mattson MP Maudsley S. Class II G protein-coupled receptors and their ligands in neuronal function and protection. Neuromolecular Med (2005) 7(1–2):3–36. 10.1385/NMM:7:1-2:003 16052036 PMC2636744

[18] Holl RW Thorner MO Leong DA. Intracellular calcium concentration and growth hormone secretion in individual somatotropes: effects of growth hormone-releasing factor and somatostatin. Endocrinology (1988) 122(6):2927–32. 10.1210/endo-122-6-2927 2453353

[19] Tsaneva-Atanasova K Sherman A van Goor F Stojilkovic SS. Mechanism of spontaneous and receptor-controlled electrical activity in pituitary somatotrophs: experiments and theory. J Neurophysiol (2007) 98(1):131–44. 10.1152/jn.00872.2006 17493919

[20] Lin-Su K Wajnrajch MP Growth hormone releasing hormone (GHRH) and the GHRH receptor. Rev Endocr Metab Disord (2002) 3(4):313–23. 10.1023/A:1020949507265 12424433

[21] Anderson LL Scanes CG. Nanobiology and physiology of growth hormone secretion. Exp Biol Med (Maywood) (2012) 237(2):126–42. 10.1258/ebm.2011.011306 22312059

[22] Stojilkovic SS Tabak J Bertram R. Ion channels and signaling in the pituitary gland. Endocr Rev (2010) 31(6):845–915. 10.1210/er.2010-0005 20650859 PMC3365841

[23] Chen C Xu R Clarke IJ Ruan M Loneragan K Roh SG. Diverse intracellular signalling systems used by growth hormone-releasing hormone in regulating voltage-gated Ca2+ or K channels in pituitary somatotropes. Immunol Cell Biol (2000) 78(4):356–68. 10.1046/j.1440-1711.2000.00917.x 10947860

[24] Muller EE Locatelli V Cocchi D. Neuroendocrine control of growth hormone secretion. Physiol Rev (1999) 79(2):511–607. 10221989 10.1152/physrev.1999.79.2.511

[25] Yang SK Wang K Parkington H Chen C. Involvement of tetrodotoxin-resistant Na+ current and protein kinase C in the action of growth hormone (GH)-releasing hormone on primary cultured somatotropes from GH-green fluorescent protein transgenic mice. Endocrinology (2008) 149(9):4726–35. 10.1210/en.2008-0405 18535104

[26] Pombo CM Zalvide J Gaylinn BD Dieguez C. Growth hormone-releasing hormone stimulates mitogen-activated protein kinase. Endocrinology (2000) 141(6):2113–9. 10.1210/endo.141.6.7513 10830298

[27] Zeitler P Siriwardana G. Stimulation of mitogen-activated protein kinase pathway in rat somatotrophs by growth hormone-releasing hormone. Endocrine (2000) 12(3):257–64. 10.1385/ENDO:12:3:257 10963046

[28] Granata R Trovato L Gallo MP Destefanis S Settanni F Scarlatti F Growth hormone-releasing hormone promotes survival of cardiac myocytes in vitro and protects against ischaemia-reperfusion injury in rat heart. Cardiovasc Res (2009) 83(2):303–12. 10.1093/cvr/cvp090 19293247

[29] Izdebski J Pinski J Horvath JE Halmos G Groot K Schally AV. Synthesis and biological evaluation of superactive agonists of growth hormone-releasing hormone. Proc Natl Acad Sci U S A (1995) 92(11):4872–6. 10.1073/pnas.92.11.4872 7761415 PMC41809

[30] Schally AV Varga JL Engel JB. Antagonists of growth-hormone-releasing hormone: an emerging new therapy for cancer. Nat Clin Pract Endocrinol Metab (2008) 4(1):33–43. 10.1038/ncpendmet0677 18084344

[31] Zarandi M Varga JL Schally AV Horvath JE Toller GL Kovacs M Lipopeptide antagonists of growth hormone-releasing hormone with improved antitumor activities. Proc Natl Acad Sci U S A (2006) 103(12):4610–5. 10.1073/pnas.0511348103 16537407 PMC1450219

[32] Kanashiro-Takeuchi RM Szalontay L Schally AV Takeuchi LM Popovics P Jaszberenyi M New therapeutic approach to heart failure due to myocardial infarction based on targeting growth hormone-releasing hormone receptor. Oncotarget (2015) 6(12):9728–39. 10.18632/oncotarget.3303 25797248 PMC4496393

[33] Lucas R Sridhar S Rick FG Gorshkov B Umapathy NS Yang G Agonist of growth hormone-releasing hormone reduces pneumolysin-induced pulmonary permeability edema. Proc Natl Acad Sci U S A (2012) 109(6):2084–9. 10.1073/pnas.1121075109 22308467 PMC3277580

[34] Letsch M Schally AV Busto R Bajo AM Varga JL. Growth hormone-releasing hormone (GHRH) antagonists inhibit the proliferation of androgen-dependent and -independent prostate cancers. Proc Natl Acad Sci U S A (2003) 100(3):1250–5. 10.1073/pnas.0337496100 12538852 PMC298759

[35] Szalontay L Schally AV Popovics P Vidaurre I Krishan A Zarandi M Novel GHRH antagonists suppress the growth of human malignant melanoma by restoring nuclear p27 function. Cell Cycle (2014) 13(17):2790–7. 10.4161/15384101.2015.945879 25486366 PMC4615138

[36] Ludwig B Ziegler CG Schally AV Richter C Steffen A Jabs N Agonist of growth hormone-releasing hormone as a potential effector for survival and proliferation of pancreatic islets. Proc Natl Acad Sci U S A (2010) 107(28):12623–8. 10.1073/pnas.1005098107 20616039 PMC2906543

[37] Schmid J Ludwig B Schally AV Steffen A Ziegler CG Block NL Modulation of pancreatic islets-stress axis by hypothalamic releasing hormones and 11beta-hydroxysteroid dehydrogenase. Proc Natl Acad Sci U S A (2011) 108(33):13722–7. 10.1073/pnas.1110965108 21825133 PMC3158163

[38] Green IC Southern C Ray K. Mechanism of action of growth-hormone-releasing hormone in stimulating insulin secretion in vitro from isolated rat islets and dispersed islet cells. Horm Res (1990) 33(5):199–204. 10.1159/000181509 1980261

[39] Bailey CJ Wilkes LC Flatt PR Conlon JM Buchanan KD. Effects of growth hormone-releasing hormone on the secretion of islet hormones and on glucose homeostasis in lean and genetically obese-diabetic (ob/ob) mice and normal rats. J Endocrinol (1989) 123(1):19–24. 10.1677/joe.0.1230019 2572664

[40] Hermansen K Kappelgaard AM. Characterization of growth hormone-releasing hormone stimulation of the endocrine pancreas: studies with alpha- and beta-adrenergic and cholinergic antagonists. Acta Endocrinol (Copenh) (1987) 114(4):589–94. 2883798 10.1530/acta.0.1140589

[41] Ludwig B Rotem A Schmid J Weir GC Colton CK Brendel MD Improvement of islet function in a bioartificial pancreas by enhanced oxygen supply and growth hormone releasing hormone agonist. Proc Natl Acad Sci U S A (2012) 109(13):5022–7. 10.1073/pnas.1201868109 22393012 PMC3324017

[42] Frohman LA Ectopic hormone production. Am J Med (1981) 70(5):995–7. 10.1016/0002-9343(81)90847-0 7234883

[43] Ghazi AA Amirbaigloo A Dezfooli AA Saadat N Ghazi S Pourafkari M Ectopic acromegaly due to growth hormone releasing hormone. Endocrine (2013) 43(2):293–302. 10.1007/s12020-012-9790-0 22983831 PMC3553305

[44] Rivier J Spiess J Thorner M Vale W Characterization of a growth hormone-releasing factor from a human pancreatic islet tumour. Nature (1982) 300(5889):276–8. 10.1038/300276a0 6292724

[45] Shibasaki T Shizume K Masuda A Nakahara M Hizuka N Miyakawa M Plasma growth hormone response to growth hormone-releasing factor in acromegalic patients. J Clin Endocrinol Metab (1984) 58(1):215–7. 10.1210/jcem-58-1-212 6417156

[46] Rorsman P Braun M. Regulation of insulin secretion in human pancreatic islets. Annu Rev Physiol (2013) 75:155–79. 10.1146/annurev-physiol-030212-183754 22974438

[47] Gilon P Chae HY Rutter GA Ravier MA Calcium signaling in pancreatic beta-cells in health and in type 2 diabetes. Cell Calcium (2014) 56(5):340–61. 10.1016/j.ceca.2014.09.001 25239387

[48] Ahren B Holst JJ. The cephalic insulin response to meal ingestion in humans is dependent on both cholinergic and noncholinergic mechanisms and is important for postprandial glycemia. Diabetes (2001) 50(5):1030–8. 10.2337/diabetes.50.5.1030 11334405

[49] Seino Y Miki T Fujimoto W Young Lee E Takahashi Y Minami K Cephalic phase insulin secretion is KATP channel independent. J Endocrinol (2013) 218(1):25–33. 10.1530/JOE-12-0579 23608222

[50] Campbell JE Drucker DJ. Pharmacology, physiology, and mechanisms of incretin hormone action. Cell Metab (2013) 17(6):819–37. 10.1016/j.cmet.2013.04.008 23684623

[51] Rajan S Dickson LM Mathew E Orr CM Ellenbroek JH Philipson LH Chronic hyperglycemia downregulates GLP-1 receptor signaling in pancreatic beta-cells via protein kinase A. Mol Metab (2015) 4(4):265–76. 10.1016/j.molmet.2015.01.010 25830090 PMC4354925

[52] Nadkarni P Chepurny OG Holz GG. Regulation of glucose homeostasis by GLP-1. Prog Mol Biol Transl Sci (2014) 121:23–65. 10.1016/B978-0-12-800101-1.00002-8 24373234 PMC4159612

[53] Wijesekara N Krishnamurthy M Bhattacharjee A Suhail A Sweeney G Wheeler MB. Adiponectin-induced ERK and Akt phosphorylation protects against pancreatic beta cell apoptosis and increases insulin gene expression and secretion. J Biol Chem (2010) 285(44):33623–31. 10.1074/jbc.M109.085084 20709750 PMC2962460

[54] Malm HA Mollet IG Berggreen C Orho-Melander M Esguerra JL Goransson O Transcriptional regulation of the miR-212/miR-132 cluster in insulin-secreting beta-cells by cAMP-regulated transcriptional co-activator 1 and salt-inducible kinases. Mol Cell Endocrinol (2016) 424:23–33. 10.1016/j.mce.2016.01.010 26797246

[55] Barabutis N Schally AV. Antioxidant activity of growth hormone-releasing hormone antagonists in LNCaP human prostate cancer line. Proc Natl Acad Sci U S A (2008) 105(51):20470–5. 10.1073/pnas.0811209106 19075233 PMC2629286

[56] Fridlyand LE Philipson LH. Does the glucose-dependent insulin secretion mechanism itself cause oxidative stress in pancreatic beta-cells? Diabetes (2004) 53(8):1942–8. 10.2337/diabetes.53.8.1942 15277370

[57] Padgett LE Broniowska KA Hansen PA Corbett JA Tse HM. The role of reactive oxygen species and proinflammatory cytokines in type 1 diabetes pathogenesis. Ann N Y Acad Sci (2013) 1281:16–35. 10.1111/j.1749-6632.2012.06826.x 23323860 PMC3715103

[58] Romero MJ Lucas R Dou H Sridhar S Czikora I Mosieri EM Role of growth hormone-releasing hormone in dyslipidemia associated with experimental type 1 diabetes. Proc Natl Acad Sci U S A (2016) 113(7):1895–900. 10.1073/pnas.1525520113 26831066 PMC4763773

[59] Fridlyand LE Philipson LH A computational systems analysis of factors regulating alpha cell glucagon secretion. Islets (2012) 4(4):262–83. 10.4161/isl.22193 23093806 PMC3496652

[60] Elliott AD Ustione A Piston DW Somatostatin and insulin mediate glucose-inhibited glucagon secretion in the pancreatic alpha-cell by lowering cAMP. Am J Physiol Endocrinol Metab (2015) 308(2):E130–43. 10.1152/ajpendo.00344.2014 25406263 PMC4297778

[61] Cryer PE. Minireview: glucagon in the pathogenesis of hypoglycemia and hyperglycemia in diabetes. Endocrinology (2012) 153(3):1039–48. 10.1210/en.2011-1499 22166985 PMC3281526

